# The interaction on hypertension between family history and diabetes and other risk factors

**DOI:** 10.1038/s41598-021-83589-z

**Published:** 2021-02-25

**Authors:** An-le Li, Qian Peng, Yue-qin Shao, Xiang Fang, Yi-ying Zhang

**Affiliations:** Jiading District Center for Disease Control and Prevention, Shanghai, China

**Keywords:** Cardiology, Diseases, Medical research, Risk factors

## Abstract

To explore the individual effect and interaction of diabetes and family history and other risk factors on hypertension in Han in Shanghai China. The method of case–control study with l:l matched pairs was used, 342 cases of hypertension and 342 controls were selected and investigate their exposed factors with face-to-face. The method of epidemiology research was used to explore the individual effect and interaction of diabetes and family history and other risk factors on hypertension. The individual effect of family history (OR = 4.103, 95%CI 2.660–6.330), diabetes (OR = 4.219, 95%CI 2.926–6.083), personal taste (OR = 1.256, 95%CI 1.091–1.593), drinking behavior (OR = 1.391, 95%CI 1.010–1.914) and smoking behavior (OR = 1.057, 95%CI 1.00–1.117) were significant (p < 0.05). But individual effect of sex, education, occupation, work/life pressure, environmental noise, sleeping time and sports habit were not significant (p > 0.05). The OR of interaction between FH and DM to hypertension was 16.537 (95%CI 10.070–21.157), between FH and drinking behavior was 4.0 (95%CI 2.461–6.502), FH and sport habit was 7.668 (95%CI 3.598–16.344), FH and personal taste was 6.521 (95%CI 3.858–11.024), FH and smoking behavior was 5.526 (95%CI 3.404–8.972), FH and work/life pressure was 4.087 (95%CI 2.144–7.788). The SI of FH and DM was 2.27, RERI was 8.68, AP was 52.48% and PAP was 55.86%. FH and DM, personal taste, smoking behavior had positive interaction on hypertension, but FH and sport habits, drinking behavior, work/life pressure had reverse interaction on hypertension. FH and diabetes were very important risk factors with significant effect for hypertension. FH and diabetes, personal taste, smoking behavior had positive interaction on hypertension, but FH and sport habits, drinking behavior, work/life pressure had reverse interaction on hypertension.

## Introduction

Hypertension is a multifactorial disease caused by genetic and acquired environmental factors, the role of gene and gene, as well as between gene and acquired environmental factors, including among acquired risk factors, leads to increased risk of hypertension and disease among different populations. Unhealthy lifestyle including obesity and lack of exercise can significantly increase hypertension incidence^1–4^. The study result from familial aggregation showed that the prevalence rate of brothers and sisters in offspring was from 20 to 66% in positive population of parents, the estimated possibility of hereditary was over 50% in a plurality of twin studies^5,6^. It showed that more than half of the blood pressure changed could be attributed to the accumulation of genetic effects.

In fact, the individual effect of single factor on the result could not truly reflect the actual effect of factors, because there was interaction between risk factors, which might weaken the role of single factor, or enhance the role of single factor. Therefore, the interaction between factors may be more able to reflect the real relationship between factors and results. There were many studies on the influencing factors of hypertension at home and abroad, but there were few reports on the interaction between the influencing factors of hypertension.

There were two models to analyze biological interaction: addition and multiplication. To explore the risk factors and etiological factors of hypertension from the perspective of environment and genetics, and to carry out effective etiological prevention are the fundamental countermeasures and measures to reduce the incidence of hypertension. It might be of public health significance to explore the interaction with additive model. Therefore, this study selected additive model to analyze the interaction between family history of hypertension and diabetes and other exposed factors on the incidence of hypertension.

## Methods

### Source of cases and controls

All cases were randomly selected from hypertension registry and follow-up management system in Jiading district in Shanghai, and all controls were randomly selected from the community population. The cases of this study were patients with hypertension who have been definitely diagnosed in the hospital and had been using antihypertensive drugs (SBP ≥ 140 mmHg or DBP ≥ 90 mmHg without using antihypertensive drugs). The controls of this study was non hypertensive patients, their blood pressure were SBP < 140 mmHg and DBP < 100 mmHg and unused antihypertensive drugs. According to l:l matched pairs design, all controls had no hypertension, and controls were required the same sex, same race, living in the same community as cases, and the difference of age was not more than 5 years old and at the same age group. Every case or control gave informed consent to participate in the study which was approved by the local ethics committee (JD-2016-KY-18). They were able to correctly respond to the investigators for the health information of themselves and their nuclear family members.

### Investigation method and content

Investigation was conducted by trained public health investigators, using a unified questionnaire. Using direct survey method, the contents of the questionnaire mainly include: age, sex, age of onset, diagnosis time, hospital name, diabetes history and so on. The criteria for judging whether all the respondents had essential hypertension and diabetes (all relatives of cases and controls): whether they had been diagnosed with essential hypertension or diabetes in the hospital before this investigation. If they had been diagnosed with essential hypertension in the hospital, it is “Yes”; if they had not been diagnosed, it is “No”.

### Statistical analysis

Statistical analyses were performed using the statistical software package (IBM SPSS statistics version 21). When *P* values < 0.05, the difference was considered statistically significant. Mean and standard deviation (SD) were used to compute for quantitative variables (age and so on), and comparisons between groups were performed by t-test. Number (n) and percentage (%)) were computed for the categorical data, comparisons between groups were performed by the chi-square (*χ*^2^) test. Multivariate logistic regression analyses were conducted for investigated risk factors, odds ratios (OR) and 95% confidence intervals (CI) were calculated. In multivariate analysis, OR were adjusted by sex. The additive model was used by cross analysis to calculate the additive interaction effect^1^. The calculated indicators were SI (synergistic effect index), RERI (relative excess risk due to interaction), AP (attributable proportion due to interaction) and PAP (the percentage of the interaction between the pure factors).

### Statement

All methods were carried out in accordance with relevant guidelines and regulations. The investigated object, content and methods of study were implemented according to the design scheme and technical route.

### Ethics approval and consent to participate

Ethical approval was granted by Jiading district center for disease control and prevention research ethics committee. All subjects gave informed consent to participate in the study, they would like to participate in investigation and answer all the related questions in the questionnaire.

## Results

### Individual effect analysis

Among 684 investigated participants (342 hypertension cases and 342 controls) aged 28–87 years old in this study, male was 50.73%, female was 49.27%. 76.17% participants had family history of hypertension, 23.83% had not. Between case group and control group, the statistical test results showed that the difference of sex, education level, work and life pressure, living environmental noise, person's taste, sleeping time, sports habit, drinking behavior and smoking behavior was no significant (p > 0.05). But the difference of family history (FH) and occupation between case group and control group was significant (p < 0.05). The difference of mean age between case group and control group was no significant (t = 0.894, p = 0.372).

The result of logistic regression analysis showed that individual effect of family history of hypertension, diabetes history, personal taste, drinking behavior and smoking behavior were significant (p < 0.05). But individual effect of sex, education, occupation, work/life pressure, environmental noise, sleeping time and sports habit were not significant (p > 0.05). See Table 1.

**Table 1:** The logistic regression analysis results of investigated risk factors on hypertension.

Variables	B	S.E.	Wals	df	Sig.	OR	OR 95% C.I.	
							Lower	Upper
Sex	0.255	0.205	1.551	1	0.213	0.775	0.519	1.157
Education	0.091	0.103	0.791	1	0.374	1.096	0.896	1.339
Occupation	0.096	0.078	1.502	1	0.220	0.909	0.780	1.059
Work/life pressure	0.156	0.155	1.023	1	0.312	1.169	0.864	1.583
Environmental noise	0.057	0.145	0.154	1	0.695	0.945	0.711	1.255
Personal taste	0.082	0.021	8.558	1	0.009	1.256	1.091	1.593
Sleeping time	− 0.260	0.142	3.344	1	0.067	0.771	0.584	1.019
Sports habit	0.090	0.125	0.512	1	0.474	1.094	0.856	1.398
Drinking behavior	0.330	0.163	4.093	1	0.048	1.391	1.010	1.914
Smoking behavior	0.080	0.028	3.788	1	0.049	1.057	1.000	1.117
Diabetes history	1.440	0.187	59.445	1	< 0.001	4.219	2.926	6.083
Family history	1.412	0.221	40.733	1	< 0.001	4.103	2.660	6.330
Constant	0.224	0.786	0.097	1	0.756	0.783		

The OR result showed that family history of hypertension, diabetes history, drinking behavior and smoking behavior were important risk factors to hypertension. The OR between family history and hypertension was 4.103 (95%CI 2.660–6.330); the OR between diabetes history and hypertension was 4.219 (95%CI 2.926–6.083); the OR between drinking behavior and hypertension was 1.391 (95%CI 1.010–1.914); the OR between smoking behavior and hypertension was 1.057 (95%CI 1.000–1.117). The OR between personal taste and hypertension was 1.256 (95%CI 1.091–1.593). See Table 1.

### Interaction analysis

#### Interaction of family history and diabetes

The effect of risk factors on the occurrence of hypertension was often not independent; they often interacted with each other and promoted the occurrence of hypertension through interaction. In order to explore the interaction between family history and diabetes, the methods of interactive effects analysis was used. Table 2 shows the result of interactive effects between family history and diabetes. The OR of interaction between family history and diabetes to hypertension was 16.537, the OR of family history and diabetes to hypertension were respectively 4.505 and 4.354. OR_(FH+DM)_ > OR_FH_ + OR_DM_. It was showed that family history and diabetes have positive interaction with hypertension.

**Table 2:** The interaction between family history and diabetes on hypertension.

Family history	Diabetes	Case	Normal	OR	95%CI
0	0	30	95	1.000	
0	1	22	16	4.354	2.086–9.076
1	0	175	123	4.505	2.866–7.084
1	1	187	36	16.537	10.070–21.157

SI_(FH+DM)_ = (16.537–1)/(4.505–1 + 4.354–1) = 2.27. RERI_(FH+DM)_ = (16.537–4.505–4.354 + 1)/1 = 8.68. AP%_(FH+DM)_ = 100*8.68/16.537 = 52.48%. PAP% _(FH+DM)_ = 100*8.68/(16.537–1) = 55.86%.

According the result of Table 3, additive model was used to calculate the additive interaction effect: the synergistic effect index (SI) of family history and diabetes to hypertension was 2.27; relative excess risk due to interaction (RERI) was 8.68; Attributable proportion due to interaction (AP) was 52.48%; and the percentage of the interaction between the pure factors (PAP) was 55.86%. The result of PAP indicated that 55.86% of hypertension was attributable to the interaction of them, when exposed to both family history and diabetes risk factors.

**Table 3:** The interaction between family history and drinking behavior on hypertension.

Family history	Drinking behavior	Case	Normal	OR	95%CI
0	0	42	84	1.000	
0	1	10	27	0.741	0.329–1.669
1	0	257	104	4.942	3.258–7.497
1	1	106	53	4.000	2.461–6.502

#### Interaction of FH and other risk factors

In order to better observe the interaction between family history (FH) and other risk factors (drinking behavior, sport habits, personal taste, smoking behavior and work/life pressure), we changed behavior from three categories (no, occasionally and regular) to two categories (yes or no), the combination of occasionally drinking and regular drinking was yes (have drink behavior); occasionally sport and regular sport was yes (have sport habits). The same as, balance taste and light taste was no salty; occasionally and regular smoking behavior was yes (smoking), little and more work/life pressure was have pressure.

The result showed OR of interaction between FH and drinking behavior to hypertension was 4.0, the OR of family history and smoking behavior to hypertension were respectively 4.942 and 0.741. OR_(FH+Dr)_ < OR_FH_ + OR_Dr_. It was showed that FH and drinking behavior have reverse interaction on hypertension. See Table 3.

The result showed OR of interaction between FH and sport habits to hypertension was 7.668, the OR of family history and sport habits to hypertension were respectively 8.571 and 1.773. OR_(FH+S)_ < OR_FH_ + OR_S_. It was showed that FH and sport habits have reverse interaction on hypertension. See Table 4.

**Table 4:** The interaction between family history and sports habits on hypertension.

Family history	Sports habits	Case	Normal	OR	95%CI
0	0	7	24	1.000	
0	1	45	87	1.773	0.716–4.395
1	0	70	28	8.571	3.586–20.487
1	1	291	131	7.668	3.598–16.344

The result showed OR of interaction between FH and taste preference to hypertension was 6.521, the OR of family history and taste preference to hypertension were respectively 4.840 and 1.386. OR_(FH+T)_ > OR_FH_ + OR_T_. It was showed that FH and taste preference (salty) have positive interaction on hypertension. See Table 5.

**Table 5:** The interaction between family history and personal taste on hypertension.

Family history	Personal taste	Case	Normal	OR	95%CI
0	0	41	93	1.000	
0	1	11	18	1.386	0.603–3.187
1	0	270	127	4.840	3.227–7.260
1	1	92	32	6.521	3.858–11.024

The result showed OR of interaction between FH and smoking behavior to hypertension was 5.526, the OR of family history and smoking behavior to hypertension were respectively 4.359 and 0.871. OR_(FH+Sm)_ > OR_FH_ + OR_Sm_. It was showed that FH and smoking behavior have positive interaction on hypertension. See Table 6.

**Table 6:** The interaction between family history and smoking behavior on hypertension.

Family history	Smoking behavior	Case	Normal	OR	95%CI
0	0	38	78	1.000	
0	1	14	33	0.871	0.417–1.816
1	0	222	105	4.359	2.820–6.739
1	1	140	52	5.526	3.404–8.972

The result showed OR of interaction between FH and work/life pressure to hypertension was 4.087, the OR of family history and work/life pressure to hypertension were respectively 5.217 and 2.229. OR_(FH+P)_ < OR_FH_ + OR_P_. It was showed that FH and work/life pressure have reverse interaction on hypertension. See Table 7.

**Table 7:** The interaction between family history and work/life pressure on hypertension.

Family history	Pressure	Case	Normal	OR	95%CI
0	0	48	107	1.000	
0	1	4	4	2.229	0.552–8.997
1	0	329	141	5.217	3.585–7.592
1	1	33	18	4.087	2.144–7.788

## Discussion

The factors influencing the occurrence of hypertension include congenital factors and natural factors, congenital factors refer to hereditary factors such as genes or family history, acquired factors mainly include bad living habits, overweight / obesity, etc. Body mass index was a comprehensive indicator of the outcome of acquired lifestyle, and closely related to the occurrence of hypertension^7–12^. Family history of hypertension was an important marker of genetic factors, it was often used as an alternative indicator to study the relationship between genetic factors and diseases^13–16^. Previous studies had not been considered the interaction between genetic and environmental factors. More attention needed to pay to the related research, to evaluate the relationship between polymorphic gene and exposed factors. Hypertension and diabetes usually occurred successively, due to the hardening of blood vessels diabetes could induce hypertension^17–19^.

There were many risk factors for hypertension, such as smoking, drinking, mental tension, lack of exercise, family genetics and so on^20,21^. In this study, the result showed that effect of family history of hypertension, diabetes history, personal taste, drinking behavior and smoking behavior were significant (p < 0.05). But effect of education, occupation, work/ life pressure, environmental noise, sleeping time and sports habit were not significant (p > 0.05). The OR result showed that family history of hypertension, diabetes history and drinking behavior were important risk factors to hypertension. The OR of family history of hypertension was 4.103, diabetes history was 4.219, drinking behavior was 1.391, smoking behavior was 1.057. This result showed that family history of hypertension, diabetes history, drinking behavior and smoking behavior were important factors of hypertension, especially family history and diabetes.

Hypertension was one of the common complications of diabetes; the incidence of hypertension in domestic diabetes patients with hypertension was 20–30%^22,23^. The OR of interaction between family history and diabetes to hypertension was 16.537. It was showed that FH and DM have positive interaction with hypertension. The percentage of the interaction between the pure factors (PAP) was 55.86%, it indicated that 55.86% of hypertension was attributable to the interaction of them, when exposed to both family history and diabetes risk factors. Because the disorder of glucose metabolism could accelerate the hardening of renal artery and systemic arteriole, increase the peripheral resistance and blood pressure, hyperglycemia can increase blood volume, overload the kidneys, retention of water and sodium, and eventually raise blood pressure. The increase of blood pressure was related to cardiac output and peripheral resistance. The increase of cardiac output without peripheral change could lead to the rise of blood pressure; the increase of peripheral resistance without the change of cardiac output or blood volume could also lead to the rise of blood pressure, and both changes of diabetic patients led to the rapid rise of blood pressure and serious complications.

Alcohol was one of the risk factors of hypertension^24–27^. Long term small amount of alcohol could increase blood pressure; small amount of alcohol could increase blood pressure, heart rate and heart load of patients with hypertension. In this study, the result of the logistic regression analysis showed that drinking behavior were risk factor to hypertension, the OR of drinking behavior was 1.391. Family history of hypertension and drinking behavior had reverse interaction on hypertension. This might be due to the interference of occasional drinking behavior, small amount of alcohol may have vascular protection. It needs further study.

High salt intake in salt sensitive individuals could lead to elevated blood pressure by affecting water and sodium metabolism, vascular function and sympathetic nervous system^28,29^. In this study, the result of the logistic regression analysis showed that personal taste were risk factor to hypertension, the OR of personal taste was 1.256. Family history of hypertension and drinking behavior had positive interaction on hypertension. The lower of 95%CI of OR < 1 (0.991), perhaps it was related to the fact that Shanghai residents generally like light food. It needs further study.

Smoking is a risk factor for cardiovascular disease, and smoking is associated with hypertension^30,31^. In this study, the result of the logistic regression analysis showed that smoking behavior were risk factor to hypertension, the OR of personal taste was 1.256. Family history and smoking behavior had positive interaction on hypertension.

Due to space limitation, only the interaction between family history and several common acquired factors were analyzed in this study. In fact, there were also interactions among acquired factors, and the interaction among multiple factors may be even more different. In short, the individual effect of single factor was strong did not mean that it must be very important role in the outcome of disease, the individual effect of single factor was weak did not mean that it must be very unimportant role in the outcome of disease. Pay attention to the interaction between factors, and expect more and better research results appear.

## Conclusion

Family history and diabetes were very important risk factors with significant effect for hypertension. FH and DM, taste preference, smoking behavior had positive interaction with hypertension, but FH and sport habits, drinking behavior, work/life pressure had reverse interaction with hypertension.

## Data Availability

The questionnaire and database supporting the conclusions of this article are available, through contact with anle_li@aliyun.com.
